# Comparative study of diversity based on heat tolerant-related morpho-physiological traits and molecular markers in tall fescue accessions

**DOI:** 10.1038/srep18213

**Published:** 2015-12-15

**Authors:** Xiaoyan Sun, Yan Xie, Yufang Bi, Jianping Liu, Erick Amombo, Tao Hu, Jinmin Fu

**Affiliations:** 1 The Key Laboratory of Horticultural Plant Genetic and Improvement of Jiangxi, Institute of Biology and Resources, Jiangxi Academy of Sciences, Nanchang 330096, China; 2 Key Laboratory of Plant Germplasm Enhancement and Specialty Agriculture, Wuhan Botanical Garden, Chinese Academy of Science, Wuhan 430074, Hubei, P.R. China; 3 Key Laboratory of High Efficent Processing of Bamboo, China National Bamboo Research Center, Hangzhou 310012, Zhejiang Province, China

## Abstract

Heat stress is a critical challenge to tall fescue (*Festuca arundinacea* Schreb.) in many areas of the globe and variations in genetic structure and functional traits is for the efficient breeding programs on developing heat tolerant cultivars. Tolerant-related morpho-physiological traits and simple sequence repeat (SSR) markers were employed to survey genetic diversity in greenhouse and growth chamber trials. 100 tall fescue accessions, including 8 commercial cultivars and 92 natural genotypes, showed a high variation in phenotypic performance under heat stress. Based on standardized heat tolerant-related morpho-physiological data, all tall fescue accessions were clustered into five groups. The accessions with similar heat tolerance were likely to be clustered in the same group. The highest genetic diversity was obtained for accessions from Africa judged by Nei’s gene diversity (0.2640) and PIC (0.2112). All grass accessions could be divided into three major groups based on SSR markers, which was partially congruous to the geographical regions and history of introduction. A low correlation was found between morpho-physiological traits and SSR markers by Mantel test. The patterns in morpho-physiological trait variations and genetic diversity associated with heat tolerance were useful to design breeding programs for developing heat stress resistance in tall fescue.

Tall fescue (*Festuca arundinacea* Schreb.) is a major cool-season grass species from the family *Poaceae*, which varies in ploidy level from diploid to dodecaploid. Tall fescue is a self-incompatible allohexaploid (2n = 6x = 42) out-crossing species containing three genomes (P, G1, and G2). It originated from Northern Europe, North Africa, Middle East, Central Asia, and Siberia, and then introduced into North and South America in about 1870s^1^. Currently, it is widely cultivated as forage and turfgrass due to its broad adaptability over a range of climatic and environmental extremes, yield, persistence, and ecosystem services. The natural tall fescue is classified into three major eco-geographic races (morphotypes): Continental, Mediterranean and Rhizomatous^2^. Using SNPs, microsatellites, ITS region, *mat K*, and phenotypic variability, genetic assessment of tall fescue germplasm showed that the morphotypes are phenotypically and genetically distinguished^3^,^4^,^5^.

High temperature is one of the major factors limiting tall fescue growth and development in transitional and warm climatic regions. High summer temperature ranging from 35 °C to 40 °C could constrain growth, inhibit photosynthesis, reduce turf quality, induce leaf withering, and even death^6^. It is expected to be a more detrimental stress in the future because of a rise in global temperature by 1–4.5 °C over the next 50 years^7^. Hence the development of improved heat tolerant cultivars of tall fescue would be a key way in alleviation of heat stress damage.

In breeding protocols, availability of information about genetic diversity among parental materials with relevant selective traits would improve the development of high stress tolerant cultivars. Genetic variability could be evaluated by both phenotypic characters (morpho-agronomic traits, physiological traits, and so on) and molecular data. Phenotypic characters are useful tools for a preliminary assessment because they could rapidly insight into the range of diversity. Several reports revealed that wild germplasm of tall fescue undoubtedly provides a large genetic variation based on phenotypic traits^3^,^8^,^9^. However, phenotypic variation may not always reflect the genetic diversity due to complex genetic nature, environmental influences, and the limited numbers^10^. Therefore, when used alone, they show some restrictions for genetic diversity studies and predicting genetic variation in breeding programs.

Molecular markers significantly reveal natural germplasm diversity of crops. This is because the markers could directly characterize the genome of organism, meanwhile they are not influenced by environment. Among the various PCR-based markers available for genetic characterization, the microsatellites or simple sequence repeats (SSRs) are widely preferred in tall fescue for diversity analysis due to their simplicity, repeatability, and high polymorphism levels^4^,^11^,^12^,^13^,^14^,^15^. However, molecular markers detected the entire genome instead of only the regions responsible for the manifestation of the traits of interest^16^. Therefore, molecular markers would be integrated with phenotypic characters to classify plant genotypes in evaluation and utilization of genetic resources, genetic diversity, and pre-breeding programs^17^.

In the present study, we surveyed genetic diversity in large accessions of tall fescue collected from different geographical regions. We used heat tolerant-related morpho-physiological traits and fluorescent dye-labeled SSR markers with better resolution of alleles in genetically heterogeneous population. Our objectives were to (i) assess genetic variation by the morpho-physiological characterizations in response to heat stress; (ii) compare and integrate the matrices of the heat tolerant-related morpho-physiological data and molecular data to assess genetic diversity of tall fescue; (iii) identify heat tolerance and sensitive accessions with distant groups for breeding prominent heat tolerant cultivars.

## Materials and Methods

### Plant materials and growth conditions

100 accessions of tall fescue including 8 turf-type commercial cultivars from the seed industry in America and 92 natural accessions from the United States Department of Agriculture-Agricultural Research Service (USDA-ARS) were selected in present study (Supplementary Table S1). The heat stress trials were implemented at Wuhan Botanical Garden, Chinese Academy of Science. All accessions were sown in petri dishes with humid papers. A single plant was randomly selected for each accession after germinating. all grasses were transplanted into plastic pots containing a mixture of sand and soil (1:1, v/v) in a controlled greenhouse (temperature ranging from 20 °C to 26 °C, 1000–1500 μmol photons m^−2^s^−1^), irrigated daily, fertilized weekly with half-strength Hoagland’s solution^18^, and mowed weekly to 7 cm canopy height. Each plant was propagated through tillers multiple times.

### Heat treatment and experimental design

A greenhouse trial and growth chamber trial were conducted in 2012. The former was processed in June, and the latter had three repeats, which implemented in August, September, and october, respectively, due to availability of two growth chambers.

1. Greenhouse trail: After breeding in the controlled greenhouse for 30 d, 100 accessions were transferred into a natural greenhouse in July 15^th^. All plots were arranged in a completely randomized block design with three replicates. The plant materials were irrigated daily until water could freely drain from the holes of plots. The temperature ranged from 39 °C to 51 °C during heat treatment period. Apart from temperature, other conditions of natural greenhouse were similar to controlled greenhouse.
2. Growth chamber trial:At each repeated trial of growth chamber, two clones of 100 accessions with 7–10 tillers were transformed into 250 mL Erlenmeyer flask wrapping with aluminum foil immersed into half-strength Hoagland’s solution. Two growth chambers during experimental period were set at 14h photoperiod, which approximately average 450 μmol photons m^−2^s^−1^, and 70% ± 10% relative humidity. All the flasks were exchanged layers daily, and the reservoir was refilled with fresh half-strength Hoagland solution every two days. After 10 d adaptation, tall fescue accessions were exposed to control (25/16 °C, day/night), and heat stress (38/30 °C, day/ night) treatments for 2 weeks. Three replications per plant were conducted in different times, and the heat treatment was subjected in different chambers for each replication.

### Morpho-physiological measurements

Heat tolerant-related morpho-physiological traits were detected every 7 d in two trails, including growth rate (GR), evapotranspiration rate (ET), turf grass quality (TQ), survival rate (SR), and leaf chlorophyll content (CHL). GR was calculated by measuring the difference of fresh weight before and after cutting on 7-day intervals. ET was measured by weight loss of the plant plot every 24 h and the relative transpiration was normalized according to a method described by Hu *et al*^19^. TQ was visually rated using a 0–9 system, in which 0 is yellow, brown or dead grass, and 9 is optimum greenness, uniformity, and dense grass according to density, texture, turf color, and smoothness. SR was assessed as a ratio between canopy survival and total plant. CHL was calculated using the method described by Hiscox and Israelstam^20^.

Each value of functional traits was standardized to the initial value, and then the average normalized Euclidean distance between different accessions was accessed following the method described by Roldan-Ruiz *et al.*^21^. All experimental data were analyzed with variance analysis (ANOVA), and the main treatment effect was separated with SPSS18.0 (IBM Corporation, New York, USA) using the traits after 2 week’s heat treatment. In addition, the heat tolerance of each accession based on functional traits was calculated following Sun *et al.*^9^ by principal component analysis (PCA) and factor analysis. The mixed models based on weighing coefficients in consideration of variance contribute of factors were constructed for two trials, respectively:


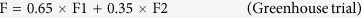



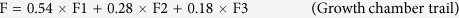


Where F was total score of heat tolerance ability of accession, F1, F2 and F3 stated factor score of principal components, respectively. The higher the F value, the better heat tolerance of accession.

### DNA isolation and SSR analysis

Before heat treatment, the fresh young leaves of each accession were collected with frozen in liquid nitrogen, and stored in a −80 °C freezer. Total genomic DNA was extracted using a cetyltrimethyl ammonium bromide (CTAB) method as described according to Doyle and Doyle^22^. Quantification was detected by a UV spectrophotometer and 0.8% agarose gel. 90 published genome-wide SSR markers^14^,^23^ were analyzed in all accessions (Supplementary Table S2). All forward primer sequences of markers were labeled with four fluorescent dyes [FAM (blue), HEX (green), TAMRA (yellow), and ROX (red)]. The PCR procedure was performed as described by Sun *et al.*^24^. All PCR reactions used a touch-down program in a 96-well My Cycler thermal cycler (Bio-Rad Inc., Hercules, CA, USA). The PCR amplified fragments were detached by an ABI 3730 DNA Sequence (Applied Biosystems Inc., Foster City, CA, USA). Alleles were labeled by GeneMarker 1.5 software (Soft Genetics, LLC, State College, PA, USA) and checked twice manually for accuracy. Because the score of individual genotype from co-dominant markers could not be distinguished unambiguously in polyploidy species, the SSR markers amplified bands of each amplified loci were entered into a binary matrix as presence (1) or absence (0) as reported by Saha *et al.*^23^

### Genetic diversity analysis

Power Marker program (a comprehensive set of statistical methods for genetic marker data analysis developed and distributed by Jack Liu, http://statgen.ncsu.edu/powermarker/index.html) was used to measure polymorphic information content (PIC) and gene diversity^25^. The phylogenetic trees were implemented using the unweighted pair group method with arithmetic average (UPGMA) based on Euclidean distance (employed heat tolerant-related morpho-physiological traits) and Neighbor-Joining tree based on shared-allele genetic distances (employed SSR markers). Bootstrapping per loci with 1000 replications was carried out to evaluate the strength of evidence for the branching patterns. And the correlation coefficients between Euclidean distance matrix based on morpho-physiological traits under heat stress and Nei’s genetic distances genetic distance matrix obtained with molecular markers was performed using the Mantel test. All analyses were conducted using PowerMarker V3.25^26^.

Three consensus trees were reconstructed by the PHYLIP program^27^ and draw by FigTree Ver.1.4.0 (a graphical viewer of phylogenetic trees and as a program for producing publication-ready figures designed by Rambaut, http://tree.bio.ed.ac.uk/software/figtree/). AMOVA implemented in the ARLEQUIN version 3.11 software^28^ was used to evaluate the population differentiation of America, European, and Asia. Significance levels of variance were assessed using 16,000 permutations.

## Results

### Variation analysis based on morpho-physiological traits

Heat stress restricted tall fescue growth, reduced the turf performance, made the leaf yellowing and wilting, and destroyed the chloroplasts (Table 1). PRGR and PRET showed relative wide variation that coefficients of variance (CV) were relative higher, while PRSR showed a narrower range of phenotypic variation in both trials. However, the CV of all functional traits was higher in growth chamber trial (Table 2). In growth chamber trial, most of tall fescue accessions had restricted growth under heat stress, but a few accessions showed allometric trend under heat stress, which the maximum of percentage relative values were more than 1, especially PRET.

The analysis of variation tendency revealed high variation among accessions that originated from different geographical regions for most of functional traits. This indicated that there was a high degree of physiological and phenotypic diversity among the accessions under heat stress. Accessions from Asia and Europe exhibited significant differences in most of functional traits under heat stress in both trials. In greenhouse trial, accessions from Asia significantly varied in PRTQ and PRSR, while those from Europe exhibited significant variability in PRSR and PRCHL. In growth chamber trial, high significant differences among accessions from Asia in PRTQ (*P* < 0.01), PRCHLT (*P* < 0.01), and PRSR (*P* < 0.01) were observed, which suggested the highest variability. In contrast, the accessions that originated from Africa showed the similar characteristic pattern of all traits under heat stress. The commercial cultivars presented less variation that just PRGR showed significant differences compared to the wild accessions, which indicated lower diversity.

### Morpho-physiological phylogenetic analysis

**Figure 1:**
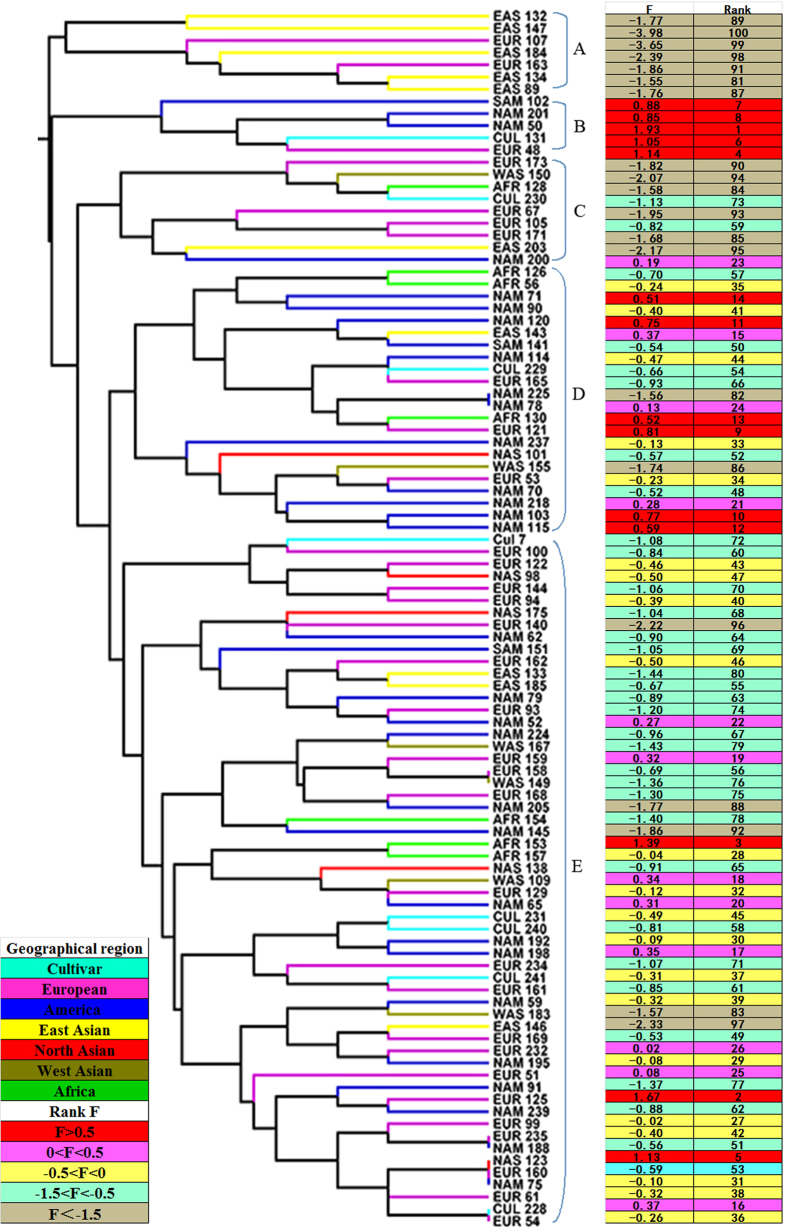
Dendrogram showing clustering pattern based on Euclidean distance from heat tolerant-related morphological data in greenhouse trial. Heat tolerance of accession was ranked based on F value on the right. F value was evaluated based on factor analysis according to the method described by Sun *et al*. (2014).

A UPGMA dendrogram of greenhouse trial was presented in Fig. 1, which separated the 100 accessions into five major groups (A–E) with a cut-off point at 0.1709. Cluster A contained 7 accessions which were characterized as heat sensitive according to the heat tolerance index (F values). Conversely, the cluster B was comprised of 5 accessions that the values of F were ranked at the top ten, showing higher heat tolerance. Cluster C contained 9 accessions, most of which were heat sensitive. Cluster D contained 22 accessions, most of which were heat tolerant. The remaining 57 accessions were grouped into Cluster E, which be defined as moderate heat tolerance.

**Figure 2:**
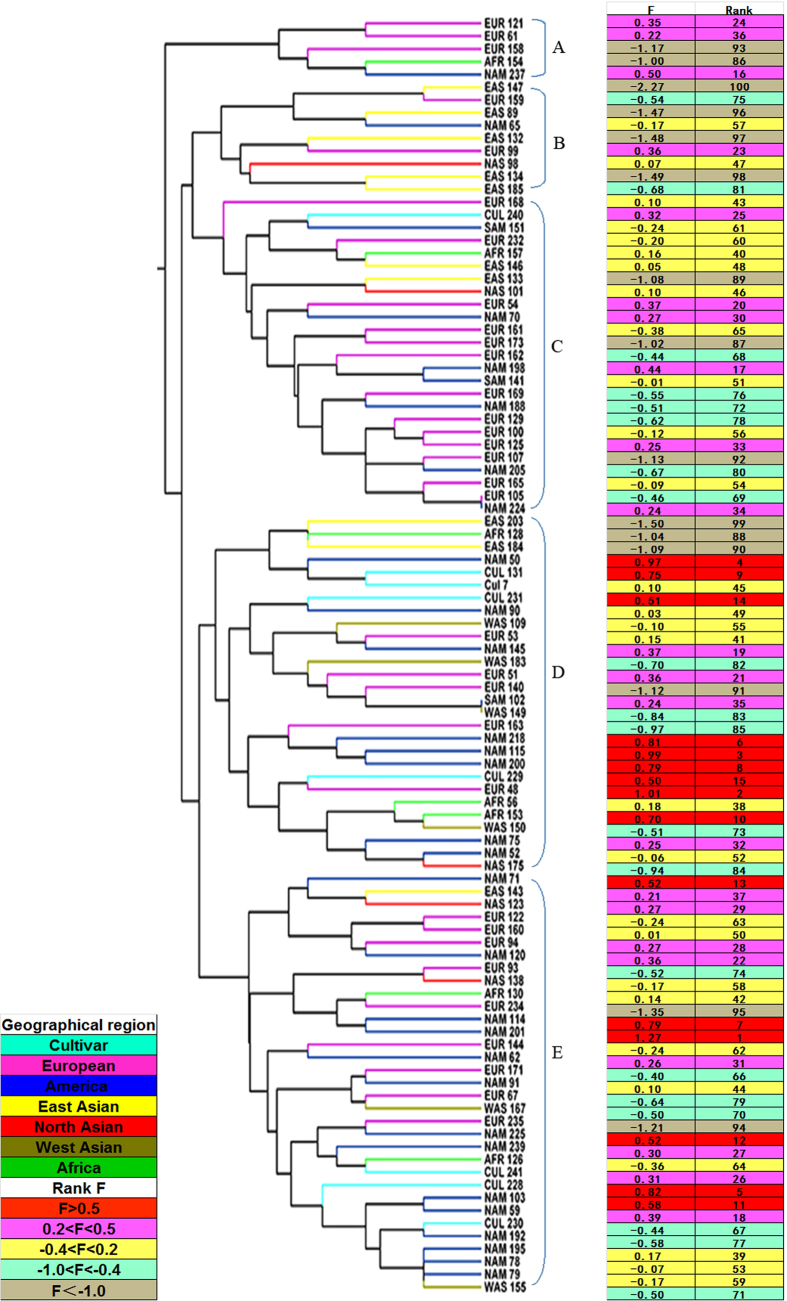
Dendrogram showing clustering pattern based on Euclidean distance from heat tolerant-related morphological data in growth chamber trial. Heat tolerance of accession was ranked based on F value on the right. F value was evaluated based on factor analysis according to the method of Sun* et al*. (2014).

Another UPGMA dendrogram based on the similarity of heat tolerant-related morpho-physiological traits in growth chamber trial also clustered into 5 groups at 0.1713 (Fig. 2). The cluster A and B were comprised of 14 accessions, most of which showed heat sensitivity. The cluster A, B and C were comprised of 39 accessions, most of which showed heat sensitivity. The cluster D and E was comprised of 61 accessions, most of which exhibited heat tolerance. Interestingly, the composition of cluster D showed complexity, in which four accessions ranked among the last ten, and another group with seven accessions ranked among the top ten. The UPGMA dendrograms of two trials were not strictly consistent to the ranking of heat tolerance based on F value by discriminant analysis. Moreover, the majority of accessions from the same geographic regions were not clustered into the same group. The accessions from Europe, Africa, and commercial cultivars showed high variation in morpho-physiological traits under heat stress, and distributed into different groups. The majority of American accessions was clustered into different groups, and exhibited high heat tolerance. However, Eastern Asian accessions showed similar heat sensitivity and therefore were clustered into the same group.

### Genetic diversity by molecular marker

**Figure 3:**
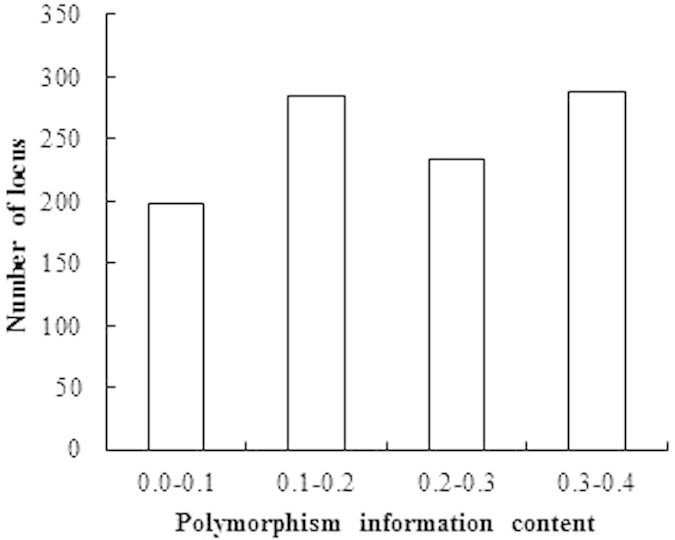
Frequency distribution of PIC for 1004 alleles of 90 SSR markers.

Genotypes of 100 tall fescue accessions generated 1204 alleles by 102 SSR markers. 1004 alleles were used to estimate the genetic diversity, which were amplified by ninety SSR markers including 13 genomic SSR markers and 77 EST-SSR with high quality genotype calls. Others that did not amplify clearly genotypic alleles were discarded. The number of alleles per microsatellite region varied from 3 (NAF114) to 26 (NFFAG018) with an average of 16.23 and 10.38 alleles per marker for genomic-SSR and EST-SSR, respectively. The polymorphism information content (PIC) value for the SSR allele ranged from 0.0164 (NFA017-176, NFA030-186, NFA030-199, NFA129-231, and so on) to 0.3750 (NFA091-165). It had an average of 0.2109, and Nei’s genetic diversity varied from 0.0165 to 0.4999 with an average of 0.2544. PIC value for genomic-SSRs allele ranged from 0.0322 to 0.3747 with an average of 0.2019, while for EST-SSRs, PIC value varied from 0.0164 to 0.3750 with an average of 0.2130. Moreover, the frequency of 93 alleles in the 1004 alleles was < 0.05, 12 alleles in which showed the least PIC value with 0.0164 (Fig. 3). 144 bands were high polymorphism alleles (PIC > 0.35), and the polymorphism of 667 alleles were moderate (0.01 < PIC < 0.35). The different polymorphism of markers would result in the varying ability to distinguish accessions.

Exploration of genetic diversity of accessions from different geographic areas was expected to have very obvious effect on conservation and utilization programs of tall fescue germplasm. The accessions from Africa showed the highest genetic diversity with Nei’s gene diversity and PIC of 0.2640 and 0.2112, followed by European (0.2448, 0.2020) and North American (0.2332, 0.1933) (Table 3). On the contrary, the lowest Nei’s genetic diversity and PIC was detected in South American (0.1571, 0.1220) and North Asian (0.1780, 0.1418). The commercial cultivars showed lower gene diversity (0.2017, 0.1633) compared with the wild accessions. Furthermore, through analysis of molecular variance (AMOVA), the genetic diversity of tall fescue accessions was only 1.87% originating from geographical populations, and almost 98.13% of variation arose from individuals within populations (Table 4).

### Cluster analysis by molecular markers

**Figure 4:**
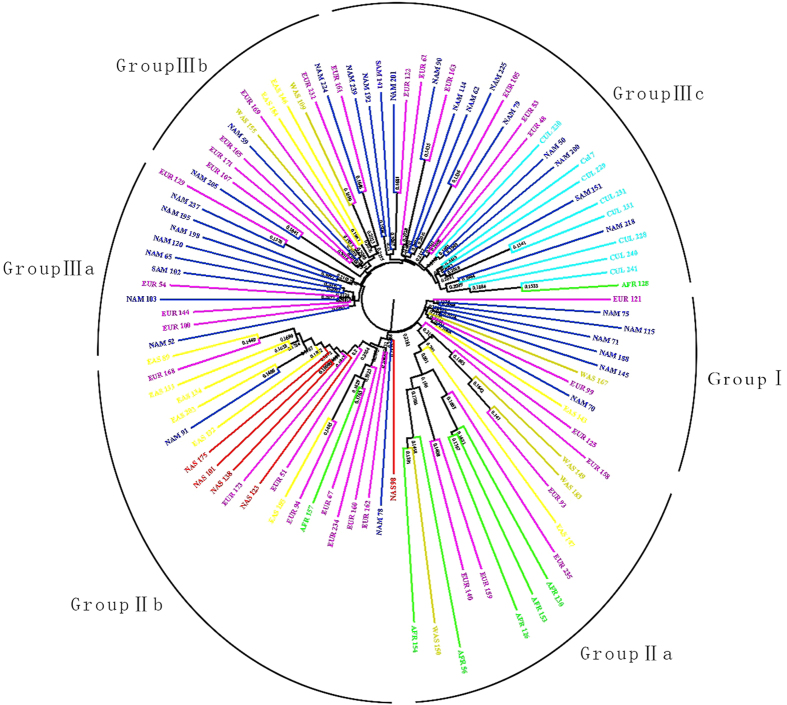
The Neighbor-Joining tree based on the shared-allele genetic distance showing clustering pattern of 100 tall fescue accessions based on SSR markers. Color dark blue indicates accession from America, color sky blue means cultivars, red means Northern Asia, yellow means Eastern Asia, yellowish brown means Western Asia, green means Africa, and purple means European.

Unrooted NJ tree based on Nei’s genetic distances from SSRs data divided 100 tall fescue accessions into three major groups (Groups I, II, III) based on the (Fig. 4). Group I contained 10 accessions from America (6), Europe (2), and Asia (2). Group II containing 37 accessions was sub-divided into two sub-clusters. The geographical differentiation was apparent. Group IIa was comprised of 15 accessions from Europe (6), Africa (5), and Asia (4), in which most of accessions collected from Africa (5/7) and half of Western Asia (3/6) were clustered. All accessions collected from Northern Asia (5/5), and most of accessions from Eastern Asia (6/10) were clustered into Group IIb, which contained 22 accessions. Groups III contained 53 accessions, which could be further divided into three subgroups: IIIa, IIIb and IIIc. Groups III was dominated by accessions from Americas (North America and South America) (24/32), and European (16/32). All commercial cultivars (8/8) were clustered into Group IIIc. Accessions from Europe were found dispersing in dendrogram.

By diagnostic nucleotides with mat*K* gene and ITS region, the distributions of tall fescue morphotypes in three groups were analysed. The majority of tall fescue accessions belonged to the Continental morphotype. The Rhizomatous morphotype including only 4 accessions dispersing in the clusters involved with other morphotypes. The dendrogram depicted that the Mediterranean morphotype may be genetically distinct from the other tall fescue morphotypes, which clustered with *F. letourneuxiana* and meadow fescue in Group II a. Inconsistent attribution were apparent for two accessions, including *F. letourneuxiana* (PI 610909) accession which clustered with Continental accessions, and a Mediterranean accessions (PI 257742) which clustered with Continental accessions. The incongruous clusters may be attributed to contaminated seeds. Further, an accession (PI 618971) collected from China that was confused to be Continental or Rhizomatous morphotype by Hand *et al.*^2^, was identified as a Continental tall fescue accessions based on the phenotypic traits and clustering analysis in present study.

### Correlations between phenotypic and molecular markers

To ascertain the extent of correspondence between genetic distances based on morpho-pysiological traits under heat stress of two trials and SSR markers, mantel matrix correspondence test was performed (Table 5). A significant positive correlation was revealed between the two morpho-physiological data sets (r = 0.116, *P* < 0.05). However, low correlations were detected between the morpho-physiological matrices and SSR data, which suggested the molecular and phenotypic classifications are discordant. The positive correlations were low but necessary, because a morphological trait was always an efficient way of routinely access several accessions in a breeding program without molecular markers.

Finally, eight heat tolerance accessions and eight heat sensitive accessions were identified based on two trials of heat stress (Table 6). Most of heat tolerant accessions selected originated from America, in contrast, the heat sensitive accessions mainly came from Eastern Asia and Spain. Except for two accessions (PI 208679 and PI 512315), the other accessions were Continental morphotype and distributed in each group of dendrogram of SSRs. Several progeny populations by controlled crossing of these accessions have been conducted in our lab to breeding programs, genetic maps, and QTLs to elucidate heat tolerance-related genes.

## Discussion

Heat stress is a major factor that inhibits growth of cool-season plants on a global scale. To relieve heat damage, identification of heat tolerant genotypes from natural germplasm would be an effective way in breeding programs. Genotypic diversity in germplasm is a prerequisite for utilizing genetic resources and successful for plant improvement in response to abiotic stress^29^. In our study, microsatellite markers and heat tolerant-related morpho-physiological traits were analyzed among 100 tall fescue accessions collected from different regions. This was done to get better understanding about the genetic diversity and identifying valuable materials for breeding heat tolerant cultivars.

### Morpho-physiological characterization

Heat tolerance of plant varies within and between species, which make it possible to identify stress tolerant germplasm due to genetic variation. Significant variation had been evaluated among tall fescue accessions for five traits in both trials. Most of accessions were damaged at varying degree by high temperature, which caused growth limitation, yellowing and withering, and even death. Conversely, many accessions from America and commercial cultivars (e.g. TF131-PureGold) exhibited excellent tolerance, superior adaption and persistence under high temperature.

In our study, the dendrograms based on morpho-physiological traits computed using genetic distance, were not precisely consistent with the ranking of heat tolerance of accessions (F value) due to different calculation patterns. However, by integrating both data, the information about regularity of distribution of heat tolerant accessions was clear and visible. Further, most of accessions with high F value had a trend to falling into one cluster, which were separated with those with lower F value falling to other cluster. The accessions were grouped into different cluster that suggested more divergence of heat tolerance than those falling in one cluster. Therefore, hybridization program with genetically diverse parents belonging to different clusters of heat tolerance would provide an opportunity for bringing heat tolerant-related genes of diverse nature together^17^,^30^,^31^.

The results of dendrograms in two trials were also incongruous, further, heat tolerant accessions exhibited different heat tolerant characterizations that indicated different tolerance mechanisms. In growth chamber trials, heat stress caused leaf evapotranspiration and withering, accelerated rate of root mortality, and reduced life span for heat sensitive accessions. Conversely, a few of heat tolerant accessions could maintain the relative higher PRGR and PRET compared to the control due to evaporative cooling effects and maintenance of carbon assimilation^32^. In greenhouse trial, all tall fescue accessions exhibited growth inhibition and evapotranspiration restraining under more than 40 °C of high temperature, as well as heat sensitive accessions showed leaf yellowing. The different mechanisms in two trials may be attributed to different stress conditions, such as soil properties and temperature^9^. In greenhouse trial, the root system temperature was buffered, and lower than shoot for property of soil. However, in growth chamber trial, the root system that was immersed into nutrition solution was directly exposed to heat stress, and was damaged. Some accessions with heat-sensitive root system showed withering synonymous to when under drought stress. Several studies suggested that roots play a critical role in plant tolerance to heat stress, and root growth is more sensitive than shoot growth to high temperatures due to their lower optimal growth temperature^33^,^34^. Xu and Huang^33^ suggested that increased soil temperature was more detrimental than high air temperature for root and shoot, and shoot growth inhibition could be induced by exposing only roots to high temperature. In contrast, plant inhibition by high air temperature could be alleviated to a great extent when the temperature of root system is decreased at a preferable level^35^. So in the growth chamber trial, the heat tolerance accessions related to heat tolerance of roots exhibited discrepant mechanism and pattern comparing with greenhouse trial.

### Molecular characterization

Microsatellites have been widely preferred in diversity analysis of tall fescue^5^,^15^,^23^. In the present study, high number of SSR alleles per locus indicated a high significant allelic richness among the panel of accessions, at a mean of 11.16 alleles per locus (ranging from 3 to 26). The result was consistent with the study of Cuyeu *et al.*^36^ that detected 14.2 alleles per locus (ranging from 5 to 24) in 161 tall fescue by analyzing 15 polymorphic SSR markers. Tehrani *et al.*^15^ obtained a low mean of 5.9 polymorphic bands ranging from 2 to 11 alleles in analysis of 37 tall fescue individuals from Iran by 11 SSR markers. Apparently, there are many factors affecting these results, such as number of accessions, origination of accessions, the number and repeat units of SSR markers.

In addition, EST-SSR exhibited similar polymorphism levels (16.23 alleles, PIC = 0.2130) to genomic-SSR (10.38, PIC = 0.2019). Its unexpected result was contrary with many reports in rice, barley, wheat, pine, and so on^37^,^38^,^39^. Part of that is because EST-SSRs are expected to be more conserved and exhibit less polymorphism than genomic-SSRs due to their location in genes. But the similar polymorphism of genomic-SSR via EST-SSR have been measured in manpolyploid species^40^,^41^, which may be associated with ploidy, taxonomy, source of SSR markers, and sampling number.

PIC value is applied to evaluate the ability of marker to distinguish the genotypes. In our study, contrary to the high alleles per locus, average Nei’s gene diversity and PIC values revealed by SSR markers were 0.2544 and 0.2109, respectively. The level of genetic diversity was similar to Tehrani *et al.*^15^ with mean of PIC (EST-SSR = 0.22, genomic-SSR = 0.27) in 37 Iranian tall fescue accessions by 11 SSRs, but was relatively lower compared to the other plant^42^. Relatively lower genetic diversity evaluated by SSR markers was expected. First, the strategy of the dominant-like score of SSR amplified bands in present study, the gene diversity and PIC of allele could not exceed 0.50. This would lead to loss of genetic information regarding heterogeneity. Secondly, the sampling strategy of single plant per accession would decrease the genetic diversity. Xu *et al.*^43^ suggested 16 randomly chosen glasses per tall fescue cultivars was the minimum number to maintain adequate genetic diversity in cultivars due to the high variation within cultivars. In addition, Tehrani *et al.*^15^ suggested that low PIC would be attributed to high predominance of unique or few alleles distributed in specific populations. In present study, a large number of unique or rare alleles with allele frequency <0.05 that amplified only in accessions from one region, were identified, and the high proportion of low PIC alleles was mostly distributed over accession from Africa and commercial cultivars (data not shown). These alleles may be attributed not only to high mutation rate of SSR markers, but also probable selection pressure of specific alleles in certain populations of tall fescue that are related to morphology and geographical environmental adaptation^29^. Interestingly, some studies also reported that the low genetic diversity may be due to the low proportion of dinucleotide SSRs^44^. In our study, only 23 out of 90 SSRs contained di-nucleotide repeats, which indicated that marker information content is related with the number of repeat units. Despite the low level polymorphism of SSRs, microsatellites data have proved to be informative enough to distinguish the relationship among wild and cultivated accessions of tall fescue.

### Divergence of genetic variation between different geographic regions

Generally speaking, higher genetic diversity would exist in the center of origin and domestication^45^. It is widely understood that tall fescue is native to much of European, the Mediterranean region including North Africa, and parts of the Middle East, and Siberia. In present study, data analysis showed that diversity among Asian and European accessions was greater than African accessions and cultivars. In contrast, the African genotypes and commercial cultivars presented less variability. In our study, both morphological traits and SSRs data revealed that natural germplasm had wide genetic diversity, while the cultivars had narrow genetic diversity because of high selection pressure and genetic drift in breeding programs^46^,^47^. All commercial cultivars used in our study from America were developed from crosses of parents that were selected from the introductions of tall fescue with narrow genetic background. Baird *et al.*^48^ reported that commercially available cultivars of turf-type tall fescue are not only similar in quality and performance, but also exhibit lower genetic diversity by DArT markers. The results indicated that neither a severe genetic bottleneck nor extensive sharing of germplasm exists in turfgrass breeding programs, and accelerating to broaden the genetic diversity in commercial germplasm of turf-type tall fescue would be put on the schedule. Moreover, it would be worthless to raise the African accessions and commercial cultivars proportion for breeding programs. Besides, most of the accessions from USDA-ARS were represented by the ones from European and North America. The discrepancies of population sizes between geographical regions would result in the variations of genetic diversity.

### Genetic clustering vs. geographic diversity

In our study, the clustering of the accessions based on heat tolerance-related morphological traits was not congruous to their geographic regions of origin. Elameen *et al.*^49^ showed that clustering based on 27 phenotypic characters could not reflect the geographic origins in 105 sweet potato accessions. Heat tolerance-related morpho-physiological traits are influenced by environmental factors, which could be attributed to genotypic variability among population. It is assumed that natural tall fescue accessions are characterized by a large adaptation. The accessions from southeast coastal regions of America had better heat tolerance with outstanding turfgrass performance under high temperature. The high ambient summer temperature due to sea-land thermodynamic property in these regions results in adaptive mechanism of tall fesuce to high temperature in summer. On the other hand, almost all accessions from Eastern Asia clustered together exhibited heat sensitivity, especially those collected from Xinjiang, where there is moderately warm summer temperature and cold in winter. All evidences supported that replaced to the tall fescue variability.

Conversely, the dendrogram based on SSRs exhibited partial geographical homogeneity within clusters. For example, most of African accessions (5/7) were grouped into Group IIa. All accessions (5) collected from Northern Asia, and most of accessions (6/10) from Eastern Asia were clustered into Group IIb. The clusterings corresponding to geographical regions of collection would be attributed to adaption to the similar environmental conditions. However, accessions from Europe and America were scattered in different groups. This might be attributed to the gene flow via germplasm exchanges among adjacent regions or history of introduction. In our study, the vast majority of accessions are Continental tall fescue, which originates from central and northern Europe eastward through a portion of Asia and is the germplasm source for most of North American tall fescue^3^. Additionally, Mediterranean tall fescue native in Northern Africa, parts of Italy and the Middle East showed infertility with Continental type^50^, and was genetically distinct from the other two morphotypes^2^,^5^. However, the Rhizomatous tall fescue as a genetically distinct third morphotype by ITS sequence and 20 SSRs^2^,^4^, was clustered with Continental type in our study. The present study had a limited number of Rhizomatous tall fescue accessions, therefore more researches need to be conducted to differentiate the three morphotypes of tall fescue. Besides, many other factors also affect the level of correlation between clustering and geographic regions, including the extent of geographic diversity, number of sampling from different geographical regions, relationship between evaluation condition and sampling regions, and so on^51^.

AMOVA suggested that 98.13% of the genetic variation resided among accessions within the population, with a statistically significant variance in heterogeneity within populations. Cuyeu *et al.*^36^ also revealed that 71% variance in 161 tall fescue accessions from 29 counties by 15 SSRs. The results indicated that tall fescue germplasm have a large diversity within the populations.

### Comparative diversity between morpho-physiological vs. molecular data

There was a positive but low correlation (Mantel’s test) between morpho-physiological matrices in two heat stress trials and molecular matrix. The lack of correlation between morphological and molecular data of tall fescue accessions might be attributed to many factors. Franco *et al.*^17^ suggested that when morpho-agronomic traits and molecular markers are utilized on a panel of genotypes for studying genetic diversity and clustering homogeneous groups, two patterns of hierarchical classifications are independently performed due to the different clustering strategies. In our study, the dendrogram based on heat tolerant-related morpho-physiological traits was constructed using a standard Euclidean distance, in which UPGMA strategy was applied. On other hand, the classification based on Nei’s distance was obtained applying Neighbor-Joining cluster strategy with SSR markers, when genetic similarities (or dissimilarities) of individuals were determined. So the formed groups based on both morpho-physiological and SSR markers had a low consistence. Sequentially, the discordance between morpho-physiological data and molecular data may be due to the difference in neutrality between the two markers. The characterized tall fescue accessions were classified according to morphological traits under heat stress conditions, which were complex and environmentally affected. The morphological traits are regulated by polygenes, which could promote plant adaptation to different environmental conditions by phenotypic plasticity and liable to subjective evaluation. In contrast, most of microsatellite variability is neutral, which would be more efficient in construction an unbiased picture of diversity and precise discrimination of closely related accessions, and the level and pattern of genetic diversity would be affected by selection, drift, and mutation. The low correlation may be also due to the low marker saturation and uneven marker distribution. More SSRs that have been uniformly mapped onto chromosomes would improve the precision of genetic diversity estimate, and is useful for seeking for quantitative trait loci (QTLs) and association mapping. Tall fescue accessions appear to be environmentally plastic, which indicated that the observed phenotypic variation under heat stress may be attributed to environmental factors to a relative large extent. The low correlations between morpho-physiological data and molecular data suggested that more SSR markers that evenly distributed on genome would be used to marker-trait association genetic studies of open-pollinated tall fescue accessions. Similar results also reported that the molecular data are not meaningfully correlated with morphological traits for several plants, such as perennial ryegrass, durum wheat, sorghum, potato, and so on^21^,^49^,^52^,^53^.

### Heat tolerant and heat sensitive accession identifying

Ali *et al.*^40^ proposed that segregation populations derived from the crosses between most diverse genotype pairs based on molecular markers and phenotypic traits would detect more informative polymorphic markers. This will be more beneficial to construct high density molecular linkage maps and QTLs compared to a population derived from the most polymorphic potential parents based on phenotype only. In our study, integration of heat tolerance-related morphological traits and F value, 8 heat tolerant and 8 heat sensitive accessions were identified for future breeding programs of outstanding heat tolerant cultivars. For the narrow genetic background of existing commercial cultivar that clustered into Group IIIc, the accessions distributing in Group I or Group II were valuable material for breeding programs. Only one accession in selected accessions is Rhizomatous tall fescue, characterized by longer and more prevalent rhizomes than other two morphotypes^3^. By overcoming the obstacle of cross-sterility with heat tolerant Continental tall fescue accessions, the excellent heat tolerant cultivars turf type with superior spreading ability and rhizomes would have been obtained. The diverse pairs of heat-tolerant accessions and their counterpart heat-sensitive could be used as potential parents for developing linkage mapping populations to detect QTLs that regulate heat tolerance in tall fescue.

## Conclusion

The present study has revealed valuable information on the functional trait variation and genetic relationships among a large number of tall fescue accessions, which will be helpful to improve heat tolerance and maintain broad genetic diversity in future tall fescue breeding programs. Although the result of both trails did not provide exactly the same description of relationships (or heat tolerance) between the tested accessions, a high proportion of accessions showed the similar level of heat tolerance. Little correspondence was also found between the morpho-physiological traits and SSR markers data. However, heat tolerant-related morphological traits and satellites are effective and efficient in quantifying and evaluating genetic diversity of tall fescue from a wide range of geographical origins. Pairs of accessions were identified that were diverse from heat tolerance and for genetic make-up, which could be used as potential parents to create mapping populations to map genes influencing heat tolerance in tall fescue. For these studied accessions are acquired from an international collections (USDA), and the high quality and easily reproducible data measured in our work could be useful to identify diverse parents for improving tall fescue heat tolerance.

## Additional Information

**How to cite this article**: Sun, X. *et al.* Comparative study of diversity based on heat tolerant-related morpho-physiological traits and molecular markers in tall fescue accessions. *Sci. Rep.* **5**, 18213; doi: 10.1038/srep18213 (2015).

## Supplementary Material

Supplementary table S1

Supplementary table S2

## Figures and Tables

**Table 1:** Descriptive statistics for growth rate (GR), turfgrass quality (TQ), survival rate (SR), evapotranspiration rate (ET), chlorophyll content (CHL) under heat stress in greenhouse trial according to geographical regions.

Geographical region	Traits	Mean	SD	Minimum	Maximum	CV/%
Cultivar	PRET	0.21	0.12	0.02	0.73	57.08
	PRGR**	0.09	0.06	0.00	0.34	64.22
	PRTQ	0.37	0.11	0.13	0.62	28.87
	PRCHLT	0.72	0.13	0.39	1.04	18.11
	PRSR	0.52	0.07	0.37	0.66	12.93
Africa	PRET	0.13	0.03	0.08	0.19	25.17
	PRGR	0.29	0.25	0.05	0.92	56.22
	PRTQ	0.36	0.14	0.08	0.64	28.70
	PRCHLT	0.67	0.17	0.25	1.00	25.16
	PRSR	0.48	0.08	0.37	0.58	16.36
Asia	PRET	0.16	0.07	0.07	0.54	46.14
	PRGR	0.24	0.13	0.00	0.68	54.57
	PRTQ**	0.26	0.13	0.06	0.50	47.83
	PRCHLT	0.53	0.16	0.05	0.83	29.94
	PRSR**	0.40	0.14	0.05	0.62	34.31
Europe	PRET	0.17	0.09	0.10	0.40	54.34
	PRGR	0.33	0.08	0.20	0.45	24.09
	PRTQ	0.31	0.14	0.08	0.05	46.19
	PRCHLT**	0.73	0.14	0.59	0.99	18.61
	PRSR**	0.44	0.13	0.08	0.65	28.56
America	PRET	0.19	0.10	0.12	0.46	53.09
	PRGR*	0.20	0.11	0.29	0.44	56.41
	PRTQ	0.33	0.12	0.17	0.50	37.22
	PRCHLT	0.59	0.12	0.40	0.77	19.80
	PRSR	0.49	0.09	0.22	0.63	18.55

* and ** indicate 0.05 and 0.01 significant levels among accessions of population based on Fisher’s protected LSD test (*P* ≤ 0.05), respectively.

**Table 2:** Descriptive statistics for growth rate (GR), turfgrass quality (TQ), survival rate (SR), evapotranspiration rate (ET), chlorophyll content (CHL) under heat stress in growth chamber trial according to geographical groups.

Geographical region	Traits	Mean	SD	Minimum	Maximum	CV/%
Cultivar	PRET	1.17	1.12	0.20	4.87	96.04
	PRGR	0.39	0.36	0.06	0.93	92.82
	PRTQ	0.61	0.25	0.13	0.92	40.19
	PRCHLT	0.79	0.45	0.03	1.95	56.24
	PRSR	0.68	0.28	0.17	0.95	41.89
Africa	PRET	0.85	1.90	0.22	6.21	140.69
	PRGR	0.15	0.12	0.01	0.83	80.98
	PRTQ	0.38	0.33	0.12	0.83	69.31
	PRCHLT	0.67	0.30	0.19	1.04	44.48
	PRSR	0.64	0.21	0.30	0.88	32.33
Asia	PRET	0.95	1.38	0.07	6.88	145.57
	PRGR	0.27	0.44	0.00	0.75	93.47
	PRTQ**	0.43	0.27	0.12	0.99	64.53
	PRCHLT**	0.71	0.36	0.03	1.52	51.34
	PRSR**	0.60	0.26	0.06	0.98	42.74
Europe	PRET	0.81	0.94	0.04	7.29	116.00
	PRGR *	0.24	0.29	0.00	0.73	77.19
	PRTQ**	0.50	0.23	0.10	0.87	45.26
	PRCHLT	0.79	0.35	0.10	1.19	44.75
	PRSR*	0.69	0.21	0.06	0.88	31.06
America	PRET	0.77	1.18	0.08	5.81	93.13
	PRGR	0.29	0.33	0.01	0.87	85.25
	PRTQ*	0.59	0.22	0.13	0.86	37.96
	PRCHLT	0.52	0.33	0.08	1.21	40.50
	PRSR	0.67	0.19	0.10	0.95	28.17

* and ** indicate 0.05 and 0.01 significant levels among accessions of population based on Fisher’s protected LSD test (*P* ≤ 0.05), respectively.

**Table 3:** Estimates of genetic parameters of 100 tall fescue accessions of different geographical regions based on 90 SSR primers.

Geographical region	Sample Size	Gene Diversity	PIC
Cultivars	8	0.2017	0.1633
Africa	7	0.2640	0.2112
Eastern Asian	10	0.2164	0.1760
Western Asia	6	0.2170	0.1746
Northern Asia	5	0.1780	0.1418
Europe	32	0.2448	0.2020
South America	3	0.1571	0.1220
North America	29	0.2332	0.1933

**Table 4:** Analysis of molecular variance (AMOVA) between Asia, Europe and America.

Source of variation	Sum of squares	Variance component	Percentage of variation (%)
Among populations	718.14	2.18^Va^	1.87
Among individuals within populations	19770.11	114.94^Vb^	98.13

**Table 5:** Correlation between dissimilarity matrices obtained with different marker types.

Row	Distance A	Distance B	Correlation	P-Value
1	Growth chamber trial	Molecular marker	0.039154727	0.223611143
2	Greenhouse trial	Molecular marker	0.017277464	0.689924675
3	Greenhouse trial	Growth chamber trial	0.11563941	0.020689413

**Table 6:** Heat tolerant and heat sensitive accessions based on heat tolerant-related morpho-physiological traits by integrity of two trials of heat stress.

	Number of Trial	ID number	Origination	Morphotype race	Cluster	Growth chamber trial		Greenhouse trial	
						F value	Rank	F value	Rank
Heat tolerance	TF48-ETC	PI 527504	France	Continental	Group IIIc	1.01	2	1.14	4
	TF50-NATM	PI 531230	USA	Continental	Group IIIc	0.97	4	1.93	1
	TF71-NATM	PI 578718	USA	Continental	Group I	0.52	13	0.51	14
	TF103-NATM	PI 578719	USA	Continental	Group IIIa	0.58	11	0.77	10
	TF115-NATM	PI 561430	USA	Continental	Group I	0.99	3	0.59	12
	TF131-Puregold	Puregold	Cultivar	Cultivar	Group IIIc	0.75	9	1.05	6
	TF153-AM	PI 208679	Algeria	F*. letourneuxiana*	Group IIa	0.70	10	1.39	3
	TF201-NATC	PI 608025	USA	Continental	Group IIIc	1.27	1	0.85	8
Heat sensitive	TF89-NASM	PI 655104	USA	Continental	Group IIb	−1.47	96	−1.76	87
	TF107-ETM	PI 512315	Spain	Rhizomatous	Group IIIb	−1.13	92	−3.65	99
	TF132-EASM	PI 618971	China	Continental	Group IIb	−1.48	97	−1.77	89
	TF140-ETM	PI 423045	Spain	Continental	Group II a	−1.12	91	−2.22	96
	TF147-EASTC	PI 499495	China	Continental	Group I	−2.27	100	−3.98	100
	TF173-ETM	PI 311044	Romania	Continental	Group IIb	−1.02	87	−1.82	90
	TF184-EASTM	PI 388897	Japan	Continental	Group IIIb	−1.09	90	−2.38	98
	TF203-EASTC	PI 632516	China	Continental	Group IIb	−1.50	99	−2.17	95
